# *Plasmodium ovale*: a case of not-so-benign tertian malaria

**DOI:** 10.1186/1475-2875-13-85

**Published:** 2014-03-10

**Authors:** Kathy-Anne Strydom, Farzana Ismail, John Frean

**Affiliations:** 1 National Health Laboratory Services, Tshwane Academic Division, Department of Medical Microbiology, University of Pretoria, Pretoria, South Africa; 2 Centre for Opportunistic, Tropical and Hospital Infections, National Institute for Communicable Diseases, Johannesburg, South Africa; 3 Wits Research Institute for Malaria, School of Pathology, Faculty of Health Sciences, University of the Witwatersrand, Johannesburg, South Africa

**Keywords:** *Plasmodium ovale*, Severe, Malaria, Infection

## Abstract

Severe malaria is most commonly associated with *Plasmodium falciparum. Plasmodium vivax* is increasingly recognized as being capable of causing severe disease. In contrast, *Plasmodium ovale* is considered as a cause of benign disease and evidence supporting the occurrence of severe or complicated ovale infection is rare. This report describes a case of severe *P. ovale* infection in a patient presenting with jaundice, respiratory distress, severe thrombocytopenia, petechiae, and hypotension. He had no apparent underlying risk factors for severe disease.

## Background

Malaria is the most important parasitic disease of man [1]. According to the World Health Organization (WHO) an estimated 3.3 billion people are at risk of malaria [2]. Annually there are over 200 hundred million clinical cases of malaria with an estimated 660,000 deaths, 90% of which occur in sub-Saharan Africa, where children under five years are most severely affected [2].

Disease in humans is caused by five species of apicomplexan parasites belonging to the genus *Plasmodium*[1]. *Plasmodium falciparum* is most commonly associated with severe disease [1-3]. Severe malaria is also known to occur with *Plasmodium vivax* and *Plasmodium knowlesi*[1,4,5]. Infection with *Plasmodium malariae* is generally benign, but has been associated with nephrotic syndrome and severe anaemia [3,6-8].

Although the clinical presentations of *Plasmodium ovale* and *P. vivax* infections largely overlap, severe disease due to *P. ovale* is extremely rare [1,3,9]. This case report describes an uncommonly severe case of ovale malaria in a patient with no apparent underlying risk factors for severe disease. The existing literature documenting severe or complicated presentations of *P. ovale* infection is reviewed.

## Case presentation

A 42-year-old male, with no significant previous medical history, was referred by a general practitioner to the Steve Biko Academic Hospital, Pretoria, South Africa. The patient presented with fever, nausea and vomiting, general body pains and shortness of breath. He complained of feeling weak and tired for the last three weeks. In the preceding six months he had worked in two malaria-endemic regions: Kalia in the north-west region of Guinea, and most recently Mozambique, from where he had returned a month previously. He had not taken malaria chemoprophylaxis during his stay in these areas.

Upon physical examination the patient was awake and alert, with no signs of meningism. He was visibly jaundiced. Abdominal examination revealed a tender right upper quadrant, there were bilateral fine crepitations on auscultation of the lungs, and petechiae were visible on his upper and lower limbs. The patient was hypotensive (blood pressure 78/58 mmHg), tachycardic (pulse rate 110 per minute) and tachypnoeic (respiratory rate 28 per minute). The temperature was 39.5°C. The chest x-ray was unremarkable.

Laboratory evaluation showed a marked thrombocytopenia (platelets 23 × 10^9^/l), mildly deranged renal function (urea 13.2 mmol/l, creatinine 157 μmol/l) and liver function tests (total bilirubin 96 μmol/l, alanine transaminase 43 U/l, aspartate transaminase 74 U/l, γ-glutamyltransferase 66 U/l). The patient had markedly elevated inflammatory markers (C-reactive protein 121.7 mg/l and procalcitonin 105.6 μg/l).

**Figure 1:**
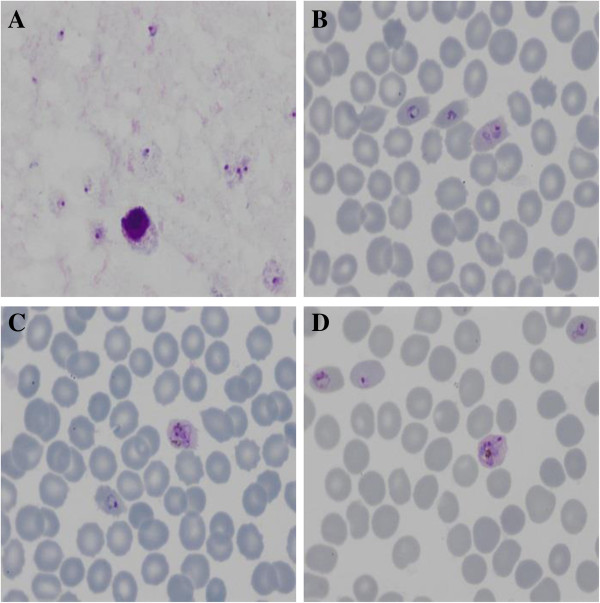
**Giemsa-stained thick and thin smears. A**. Trophozoites of *P. ovale* in a Giemsa-stained thick smear. **B**. Trophozoites and **C**, **D**. Trophozoites and immature schizonts of *P. ovale* in a Giemsa-stained thin film. Note slightly enlarged, fimbriated and oval-shaped infected red blood cells, with James’ dots.

The diagnosis of malaria was made by microscopic examination of Giemsa-stained blood smears, showing characteristic *P. ovale* parasites (Figure 1). The parasitaemia was 1.4%. Rapid diagnostic tests for *P. falciparum* antigen (histidine-rich protein 2) were repeatedly negative. Mixed *Plasmodium* species infection was excluded and the diagnosis of *P. ovale* malaria was confirmed by multiplex PCR [10,11].

The patient was admitted to high care and treated with a course of intravenous quinine (600 mg eight hourly) and doxycycline (100 mg twelve hourly). Ceftriaxone was added to cover for possible bacterial sepsis. The septic screen investigations, which comprised two sets of blood cultures and a urine culture, were negative. The timing of the blood cultures in relation to antibiotic administration is unclear.

The patient demonstrated a good clinical response to treatment, and he was stepped down to a general ward. Subsequent laboratory evaluations showed an improvement in full blood count, renal and liver functions as well as inflammatory markers, which returned to normal before discharge. Primaquine, 30 mg orally for 14 days was administered to eradicate hypnozoites and prevent possible relapses.

## Discussion

Endemic transmission of *P. ovale* is traditionally described as limited to sub-Saharan Africa and the islands of the western Pacific [9]. Infections with *P. ovale* have also been documented from India, the Middle East and parts of Southeast Asia [6,9]. Two non-recombining sympatric forms of *P. ovale* occur globally [12]. *Plasmodium ovale curtisi* (classic type) and *Plasmodium ovale wallikeri* (variant type) have been proposed as two distinct species [12,13].

Much of what is currently known regarding the epidemiology of *P. ovale* is based upon surveys utilising light microscopy as diagnostic tool [6]. From these surveys, the prevalence of *P. ovale* is generally considered to be low and ranges between 3-5% and greater than 10% in areas of West and Central Africa [6]. The utility of light microscopy is limited by difficulties in distinguishing between *P. ovale* and *P. vivax* in smears, as well as the low parasitaemias characteristic of *P. ovale* infection. In addition, immunochromatography-based rapid diagnostic tests display poor sensitivity for the detection of *P. ovale* infection [14,15]. This may lead to underestimating the true burden of disease as is evident when more sensitive diagnostic modalities, such as PCR-based methods targeting small subunit rRNA, are employed [6].

*Plasmodium ovale* is known to cause mild disease with a low parasitaemia [6,9]. Literature describing severe or complicated cases of *P. ovale* infection is limited*.* These rare reports include six cases complicated by acute respiratory distress syndrome (ARDS)(one of which further complicated by renal failure and metabolic acidosis), two cases of splenic rupture, and a single case of splenic infarction [16-25]. Clinical and therapeutic data for these cases are shown in Table 1.

**Table 1:** **Summary of published cases of severe and complicated *P. ovale *** **infection**

**Reference**	**Patient age**	**Gender**	**Medical history**	**Travel history**	**Prophylaxis**	**Time to presentation**	**Parasitaemia**	**WHO or other severity criteria**	**Treatment**	**Outcome**
This case report	42 yr	Male	None	Kalia, Guinea: 6 months ago Mozambique: 1 month ago	None	1-6 months	1.4%	Jaundice, respiratory distress, hypotension, incipient bleeding	IV quinine, 14 days primaquine	Recovered
Lee *et al. *[16]	31 yr	Female	None	Ghana	Mefloquine	10 months	0.1%	ARDS	Chloroquine, 14 days primaquine	Recovered
Rojo Marcos *et al. *[17]	43 yr	Male	Hypertensive, diabetic	Nigeria	None	N/S	6,000/μL	ARDS	Chloroquine, 14 days primaquine	Recovered
Haydoura *et al. *[18]	46 yr	Female	Methylenetetrahydrofolate reductase mutation with secondary portal vein thrombosis	Acquired by transfusion	N/A	1 month following transfusion	1.11%	ARDS	IV quinine and doxycycline, 14 days primaquine	Recovered
Roze *et al. *[19]	24 yr	Male	Tuberous sclerosis	Chad, Ivory Coast	Doxycycline	1 year	0.1%	ARDS	Chloroquine then changed to quinine	Recovered

Lau *et al. *[20]	59 yr	Male	None	Victoria Island, Nigeria	Mefloquine	6 months	0.18%	ARDS, acute renal failure, metabolic acidosis	Chloroquine plus primaquine, changed to quinine, then artesunate	Demised
Hashimi *et al. *[21]	31 yr	Male	Previous pulmonary tuberculosis (20 years ago)	Democratic Republic of Congo	N/S	7 months	0.2%	ARDS	IV quinine	Demised
Facer *et al. *[23]	51 yr	Female	N/S	Ghana	None	12 days	1.8%	Splenic rupture	None	Demised
Patel *et al. *[24]	42 yr	Male	N/S	South and Central Africa	Hydroxychloroquine, discontinued during travel	18 months	N/S	Splenic rupture	Chloroquine and primaquine	Recovered
Cinquetti *et al. *[25]	34 yr	Male	None	Senegal 2002, Ivory Coast 2004	Doxycycline	2-4 years	0.001%	Splenic infarction	IV quinine	Recovered

N/S: Not stated; N/A: not applicable; ARDS: Acute respiratory distress syndrome.

The current WHO treatment guidelines for severe malaria recommend intravenous (IV) artesunate for the treatment of severe malaria due to all *Plasmodium* species [26]. Intravenous artesunate is currently not registered in South Africa for clinical use and is only available for named patients on application under Section 21 of the Medicines and Related Substances Act, usually at selected sentinel hospitals through the current artesunate access programme. The patient discussed in this case report was treated with IV quinine, which according to the current South African treatment guidelines, is still the treatment of choice for severe malaria in adult patients if IV artesunate is not readily available [27].

The pathophysiology of *P. falciparum* as the leading cause of severe malaria has been examined extensively. Various parasite, host, geographic and social factors contribute to severe disease manifestations; however, sequestration of mature parasitized red blood cells is considered to be the key pathogenic event [28,29].

*Plasmodium vivax,* long considered to cause benign infection, is increasingly recognised as a cause of severe malaria [30,31]. Similar to *P. falciparum* infection, multiple factors contribute to severe disease [30]. Evidence is emerging that *P. vivax* infected red blood cells can also cytoadhere and sequestrate in the microvasculature, but to a lesser extent than *P. falciparum *[32]. It remains to be elucidated to what degree sequestration contributes to severe disease manifestations in vivax infections [30,32].

The pathophysiological correlates and risk factors for severe *P. ovale* infection are not yet fully established. Reports of severe ovale infection remain rare; however, when the diagnostic difficulties both in the detection of a low parasitaemia and distinguishing *P. ovale* from *P. vivax,* based on traditional light microscopy is taken into account, severe cases of ovale malaria may actually be underreported.

## Consent

Verbal consent was obtained from the patient; however, due to his unavailability, written consent could not be obtained. Thus, ethical approval was obtained from the University of Pretoria Ethics Committee. The letter of approval from the Committee is available for review.

## Competing interests

The authors have no competing interests to declare.

## Authors’ contributions

KA liaised with clinician regarding management of patient, collection of clinical information, drafting of manuscript. FI reviewed manuscript. JF reviewed manuscript, confirmed identification of *P. ovale* and gave final approval for publication. All authors read and approved the final manuscript.
